# COVID-19-associated Pulmonary Aspergillosis in Mechanically Ventilated Patients at 7 US Hospitals: Epidemiology and Estimated Likelihood of Invasive Pulmonary Aspergillosis—Results of the Prospective MSG-017 Study

**DOI:** 10.1093/ofid/ofaf331

**Published:** 2025-07-17

**Authors:** M Hong Nguyen, Sixto M Leal, Luis Ostrosky-Zeichner, Andrej Spec, George R Thompson, Thomas F Patterson, John Baddley, Rachel McMullen, Drashti Shah, Cornelius J Clancy, Gerald McGwin, Peter G Pappas

**Affiliations:** Department of Medicine, Division of Infectious Diseases, University of Pittsburgh, Pittsburgh, Pennsylvania, USA; Department of Pathology, Division of Laboratory Medicine, University of Alabama at Birmingham, Birmingham, Alabama, USA; Department of Medicine, Division of Infectious Diseases, University of Texas Health Medical School, Houston, Texas, USA; Department of Medicine, Washington University, St Louis, Missouri, USA; Department of Medicine, University of California- Davis, Sacramento, California, USA; Department of Medicine, Division of Infectious Diseases, University of Texas Health Science Center at San Antonio, San Antonio, Texas, USA; Department of Medicine, Division of Infectious Diseases, Johns Hopkins University School of Medicine, Baltimore, Maryland, USA; Mycoses Study Group Central Unit, Division of Infectious Diseases, University of Alabama at Birmingham, Birmingham, Alabama, USA; Mycoses Study Group Central Unit, Division of Infectious Diseases, University of Alabama at Birmingham, Birmingham, Alabama, USA; Department of Medicine, Division of Infectious Diseases, University of Pittsburgh, Pittsburgh, Pennsylvania, USA; Department of Epidemiology, School of Public Health, University of Alabama at Birmingham, Birmingham, Alabama, USA; Mycoses Study Group Central Unit, Division of Infectious Diseases, University of Alabama at Birmingham, Birmingham, Alabama, USA; Department of Medicine, Division of Infectious Diseases, University of Alabama at Birmingham, Birmingham, Alabama, USA

**Keywords:** COVID-19-associated aspergillosis (CAPA), diagnostic criteria of CAPA, spectrum of diseases of CAPA

## Abstract

**Background:**

There is no prospective, US multicenter study of COVID-19–associated pulmonary aspergillosis (CAPA). CAPA definitions do not differentiate invasive aspergillosis (IPA) from colonization. Validity of single mycologic test results is unclear.

**Methods:**

We performed a prospective 7-center US study of mechanically ventilated adults with COVID-19 (April 2021–May 2022). Mycoses Study Group (MSGERC) CAPA criteria include host and clinical factors, imaging and test results (histopathology; bronchoalveolar lavage [BAL] culture and/or BAL or serum galactomannan-immunoassay). Proven, putative, and unlikely IPA were defined by clinical criteria. CAPA-unlikely IPA criteria included survival or negative autopsy following no/limited antifungal treatment. IPA likelihood was estimated using sensitivity/specificity of tests from autopsy data.

**Results:**

CAPA incidence was 7% (14/212). Independent CAPA risk factors were EORTC/MSGERC host factor and cavitary lesions. Seven percent, 79%, and 14% of CAPA patients had proven, putative, and unlikely IPA, respectively. Respective estimated IPA likelihoods were 84%, 7%–99%, and 1%–8%. Overall, median estimated IPA likelihood was 30%. Patients with CAPA-unlikely IPA had a single positive BAL galactomannan-immunoassay with other negative tests. CAPA mortality (71%) was not impacted by antifungal treatment or significantly different than without CAPA. CAPA incidence was 10% and 16% by European Confederation of Medical Mycology and Public Health Wales definitions, respectively. IPA was unlikely in 75% (6/8) and 57% (13/23) diagnosed by these definitions but not MSGERC.

**Conclusions:**

CAPA is associated with high mortality, but IPA's contribution is unclear. Single positive tests are insufficient for diagnosing CAPA-IPA. IPA likelihood is best estimated by combining test results (both positive and negative).

## Teaser

COVID-19–associated pulmonary aspergillosis (CAPA) incidence was 7% in US intensive care units. CAPA represents a continuum encompassing invasive pulmonary aspergillosis (IPA) and noninvasive colonization. Median estimated likelihood of IPA in patients with CAPA was 30%. Acceptance of single positive mycologic test results likely over-estimates IPA.

COVID-19–associated pulmonary aspergillosis (CAPA) is diagnosed by a constellation of host and clinical factors, imaging findings, and mycologic test results. Most COVID-19 patients lack traditional European Organization for Research and Treatment of Cancer (EORTC)/Mycoses Study Group Education and Research Consortium (MSGERC) host factors for aspergillosis, clinical and imaging findings of CAPA are difficult to distinguish from those of severe COVID-19, and histopathologic confirmation of CAPA is typically lacking. Therefore, diagnosis is driven primarily by positive *Aspergillus* culture and/or biomarkers [1, 2]. Unfortunately, bronchoalveolar lavage (BAL) culture and *Aspergillus* biomarkers are detection tests rather than definitive diagnostics. False-positive results for invasive pulmonary aspergillosis (IPA) are well-recognized in patients with severe COVID-19, especially single positive results [3–5]. Clearly, IPA is responsible for serious disease and death in some patients with COVID-19 [6–8]. However, there are reports of patients with CAPA who survived in absence of antifungal treatment or in whom autopsies were negative for IPA [1]. Other patients had confirmed IPA at autopsy, but findings were incidental given the extent of lung destruction from COVID-19 [7, 9, 10]. CAPA is best understood as the likely presence of *Aspergillus* in the respiratory tract of patients with severe COVID-19, encompassing a continuum that spans IPA and noninvasive colonization [6, 7, 10].

Diagnostic criteria for CAPA have been proposed by expert panels [11, 12], but there is no universally accepted, validated disease definition. CAPA incidence at individual hospitals has ranged from 0 to >30% among critically ill patients with COVID-19, with higher rates generally reported in those who are mechanically ventilated and with more frequent testing of BAL samples [10, 13]. Patients with CAPA have high mortality rates, which in some studies exceed that of patients with severe COVID-19 without CAPA [1, 14]. However, it is unknown how often deaths are attributable to *Aspergillus* rather than underlying COVID-19 or other causes [7, 9]. Furthermore, there is no conclusive evidence that antifungal treatment improves CAPA outcomes. At present, there is considerable uncertainty about the incidence and clinical significance of CAPA, and rates of IPA in patients with CAPA [13, 15].

There is no prospective, multicenter study of CAPA from North America. Our primary objective was to determine the epidemiology, treatment and outcomes of CAPA in US intensive care units (ICUs). Secondary objectives were to estimate the likelihood of IPA among patients diagnosed with CAPA and to compare different diagnostic definitions.

## METHODS

We enrolled adults ≥18 years old with respiratory failure from SARS-CoV-2 infection who required mechanical ventilation for ≥72 hours. Subjects were enrolled if they or their surrogates provided assent. Subjects were excluded if life expectancy was <72 hours, weaning from mechanical ventilation was expected within 24 hours, or *Aspergillus* cultures or biomarkers were not collected. Enrollment was from April 2021 through May 2022 at the University of Alabama at Birmingham (UAB), University of Pittsburgh, Washington University in St. Louis, University of California–Davis, University of Texas at Houston, University of Maryland, and University of Texas Health San Antonio.

### Patient Consent Statement

Prior to enrollment, a signed assent form was obtained from the patients or their legally authorized representatives. The study design was approved by the institutional review board at each participating center and conforms to the standards currently applied in the United States.

Clinical and radiologic data were captured by chart review. All patients were followed until hospital discharge or death. Patients who survived until discharge were followed for survival for 90 days postdischarge using the electronic medical record. BAL or nondirected bronchial lavage (NBL)/tracheal aspirate cultures, and/or BAL and serum galactomannan immunoassays (GM-EIA) were ordered at clinicians’ discretion and performed locally. All available remnant samples were shipped to a central laboratory at UAB for *Aspergillus* PCR (AsperGenius, PathoNostics) or GM lateral flow assays (GM-LFA, IMMY), which were performed weekly. Based on UAB validation studies, positive polymerase chain reaction (PCR) cycle threshold (C_T_) was ≤33 for *Aspergillus* species and *A terreus*, and ≤30 for *A fumigatus*. Positive GM-LFA index was ≥0.5 (reported as positive or negative). Results were communicated to sites immediately. Management decisions were at clinicians’ discretion.

CAPA definition. MSGERC CAPA definition was adapted from European Confederation of Medical Mycology-International Society of Human and Animal Mycology (ECMM-ISHAM) and Public Health Wales-Mycoses Research Council (PHW-MRC) definitions [Supplementary Methods 1] [11, 12]. CAPA diagnostic criteria were: (1) worsening signs and symptoms as assessed by the management team and/or site investigators; (2) pulmonary infiltrates on chest imaging; and (3) ≥ 1 of the following: BAL GM-EIA index ≥1.0, serum GM-EIA index ≥0.5, positive BAL culture.

**Figure 1.**
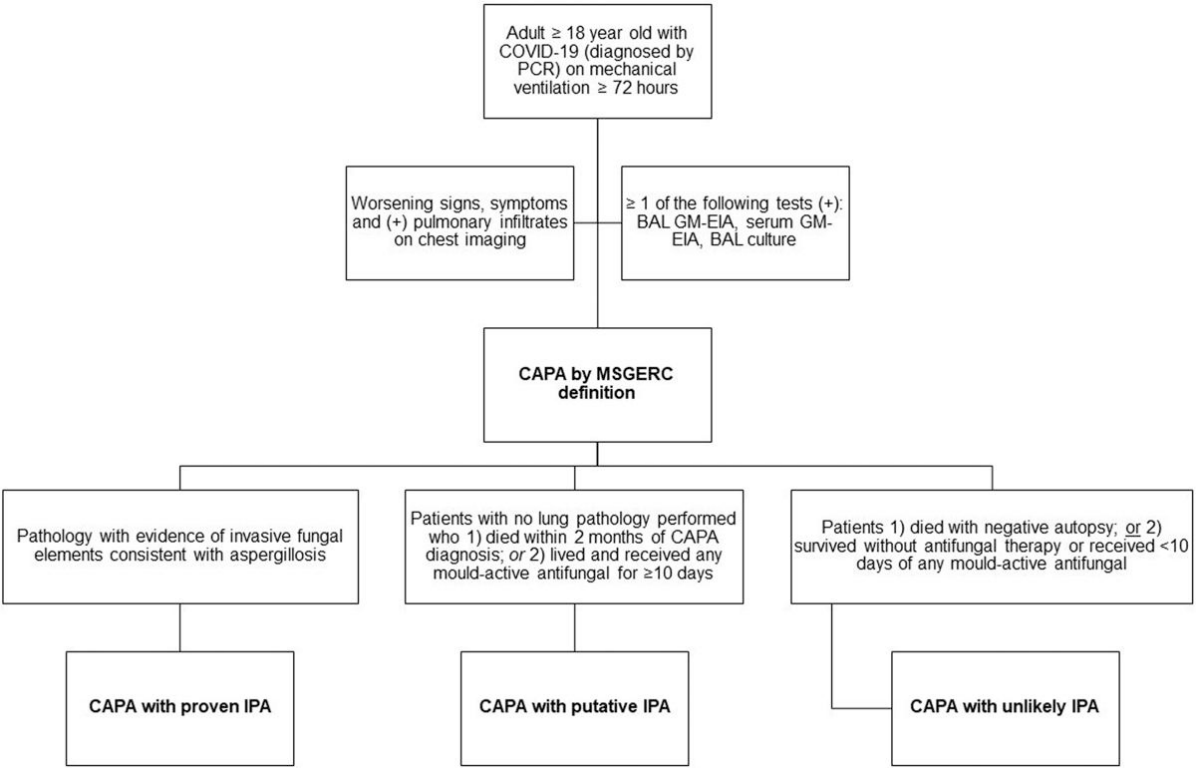
MSGERC definitions of CAPA, CAPA with proven IPA, CAPA with putative IPA, and CAPA with unlikely IPA. Abbreviations: CAPA, COVID-19–associated pulmonary aspergillosis; GM-EIA, galactomannan enzyme immunoassay; IPA, invasive aspergillosis; MSGERC, Mycoses Study Group Education & Research Consortium.

CAPA-IPA definition. CAPA was classified based on clinical criteria as CAPA with proven IPA, putative IPA, or unlikely IPA [Figure 1].

Outcomes. Primary outcomes were CAPA incidence by MSGERC definition and 90-day mortality of patients with and without CAPA. Secondary outcomes were risk factors for CAPA or mortality.

Estimation of IPA likelihood. Detailed methods and calculations for each case are provided in Supplementary Methods 2. We estimated IPA likelihood using performance (sensitivity, specificity) of BAL culture, BAL GM-EIA, and/or serum GM-EIA, as published for autopsy-proven IPA in patients with antemortem diagnoses of CAPA or influenza-associated-pulmonary aspergillosis [6]. Because *Aspergillus* PCR and GM-LFA performance have not been validated for CAPA or tissue-proven CAPA-IPA, we used sensitivity and specificity from studies of hematologic malignancy and/or other EORTC/MSGERC at-risk populations. For the first diagnostic test result in each case, we assigned 10% pretest CAPA likelihood among patients with severe COVID-19 based on median published prevalence [1]. The calculated likelihood was then used as pretest likelihood for the next test result. This process was repeated for all test results to determine the final estimated likelihood.

Statistical analysis. Data were analyzed using Stata v15 (StataCorp) and GraphPad Prism v8.0 (GraphPad Software). To more accurately estimate time to event, cumulative incidence function was performed to address death as a competing event. For assessment of mortality risk factors, patients with and without CAPA were compared using Mann-Whitney *U* (continuous variables) and Fisher exact tests (categorical variables). Variables significant by univariate analysis (*P* ≤ .05) were entered into a binary logistic regression model to determine independent risk factors for CAPA. For risk factors for mortality, Cox regression analysis with CAPA as a time-dependent variable was performed. *P* ≤ .05 (2-tailed) was significant.

## RESULTS

Two hundred-and-twenty-one critically ill patients with COVID-19 were enrolled. Nine patients were excluded (rescinded assent, n = 2; lack of CAPA testing, n = 7). The remaining 212 patients were from centers 1 (n = 73), 2 (n = 63), 3 (n = 29), 4 (*n* = 25), 5 (n = 11), 6 (n = 7), and 7 (n = 4). Median age was 53 years; 61% (129/212) of patients were men [Table 1]. Fifty-seven percent (120/212), 27% (57/212), and 15% (32/212) required prone ventilation, extracorporeal membrane oxygenation, and inhaled nitrous oxide therapy, respectively. Ninety percent (191/212) and 19% (41/212) of patients received systemic corticosteroids and tocilizumab, respectively. There were no significant differences between centers in patients’ underlying diseases or EORTC/MSGERC host risk factors.

**Table 1.** Clinical Characteristics of Critically Ill Patients, Mechanically Ventilated With COVID-19, Stratified by Presence or Absence of MSGERC-defined CAPA

Characteristics	All Patients (n = 212)	CAPA (n = 14)	No CAPA (n = 198)	*P* Value*
Demographics and underlying diseases				
Men	61% (128/211)^a^	43% (6)	62% (122/197)^a^	.17
Age, median (IQR), y	53 (21)	49 (23)	53 (31)	.8
White race	74% (135/183)^a^	67% (8/12)^a^	74% (127/171)^a^	.28
BMI, median (IQR), kg/m^2^	33.2 (10.5)	32.2 (8.9)	33.4 (10.8)	.3
COPD	12% (25)	12% (3)	11% (22)	.22
Liver cirrhosis	4% (9)	7% (1)	4% (8)	.47
Diabetes mellitus	34% (72)	21% (3)	35% (69)	.39
Hemodialysis on ICU admission	15% (31)	50% (7)	12% (24)	**.001**
EORTC/MSGERC host factors	20% (42)	43% (6)	18% (36)	**.04**
Solid organ transplant	17% (36)	43% (6)	15% (36)	**.02**
Lung, n	6	0	6	
Kidney, n	23	5	18	
Liver, n	4	1	3	
Heart, n	3	0	3	
Pancreas, n	3	1	2	
Hematological cancer	2% (5)	0%	2% (5)	1.0
Allogeneic HSCT	0.5% (1)	0%	2.0% (1)	1.0
Neutropenia >10 d	1.5% (3/194)^a^	0% (0/11)^a^	2% (3/183)^a^	1.0
Receipt of immunosuppressive agents				
T- or B-cell immunosuppressants	30% (64)	43% (6)	29% (58)	.37
Tocilizumab during hospital stay	19% (41)	14% (2)	20% (39)	1
Any IL-6 inhibitor	24% (52)	29% (4)	24% (48)	.75
Baricitinib	15% (31)	21% (3)	14% (28)	.44
Systemic corticosteroid	90% (191)	100% (14)	89% (177)	.37
Inhaled corticosteroid	17% (32/191)^a^	8% (1/13)^a^	17% (31/178)^a^	.7
Chest imaging findings				
Cavitary lesions	5% (11)	21% (3)	4% (8)	**.03**
Consolidation	42% (88)	64% (9)	40% (79)	.09
Ground glass opacities	72% (152)	57% (8)	43% (6)	.23
Worsening findings on chest CT	23% (48)	21% (3)	23% (45)	1.0
Patchy distribution	28% (60)	14% (2)	29% (58)	.36
ARDS	21% (45)	21% (3)	21% (42)	1.0
Receipt of support in ICU				
Duration of MV, median (IQR), d	20 (12)	21 (17)	20 (24)	.81
Requirement of prone ventilation (PV)	57% (120)	50% (7)	57% (113)	.78
Duration of PV, median (IQR), d	0 (3)	0 (4)	0 (6)	.99
Administration of nitrous oxide (NO)	15% (32)	29% (4)	14% (28)	.24
Duration of NO, median (IQR), d	14 (18.5)	7 (7)	15 (24)	.3
Requirement of ECMO	27% (57)	14% (2)	28% (55)	.39
Duration of ECMO, median (IQR), d	30.5 (34.5)	56.5 (59)	30.5 (35)	.39
Outcome				
ICU mortality	54% (113)	71% (10)	52% (103)	.42
90-d mortality	55% (116)	71% (10)	54% (106)	.58
Duration of ICU stay, median (IQR), d	25 (25)	27 (22)	25.5 (26)	.98
Duration of hospital stay, median (IQR), d	28 (31)	27 (22)	31 (35)	.42

Abbreviations: ARDS, acute respiratory distress syndrome; BMI, body mass index; CAPA, COVID-19–associated pulmonary aspergillosis; COPD, chronic obstructive pulmonary disease; CT, computed tomography; d, days; ECMO, extracorporeal membrane oxygenation; EORTC, Organization for Research and Treatment of Cancer; HSCT, hematopoietic stem cell transplant; ICU, intensive care unit; IFI, invasive fungal infection; IL, interleukin; IQR, interquartile range; MSGERC, Mycoses Study Group Education and Research Consortium; MV, mechanical ventilation.

We use multivariable logistic regression with binary outcome (CAPA or no CAPA) to evaluate 3 factors that were significant by univariate analyses (*P* < .05): cavitary lung lesion in chest radiograph, solid organ transplant, and requirement for hemodialysis. We identified cavitary lung lesion *(P* = .002; OR: 13.5; 95% CI, 2.6–67.3) and EORTC/MSGERC host factor (*P* = .005; OR: 6.2; 95% CI, 1.7–22.2) to be independent risk factors for CAPA.

^a^Missing data.

^*^Bolded values denote statistical significance at *P* < .05.

Chest radiographs and computed tomography scans were performed in all and 72% (153/212) of patients, respectively. Ground glass opacity (73%, 152/212) and consolidation (42%, 88/212) were most common findings. Cavitary lesions were observed in 5% (11/212). Eighty-nine percent (189/212) and 26% (56/212) fulfilled radiographic criteria for CAPA proposed by ECMM-ISHAM and PHW-MRC, respectively [Supplementary Methods 1] [11, 12].

Fifty-two percent of patients (110/212) underwent ≥ 1 diagnostic bronchoscopy and testing of BAL by cultures (83 patients, 243 samples) and/or GM-EIA (60 patients, 92 samples). Fifty-two percent (111/212) of patients had NBL/tracheal aspirate cultures; 39 patients subsequently had BAL fungal culture and/or GM performed, and 72 patients did not. Overall, 86% (182/212) of patients had ≥1 respiratory sample (BAL or NBL/trach aspirate) tested for *Aspergillus.* Seven percent (14/212) of patients were diagnosed with CAPA by MSGERC definition (range by center: 0%–27%) [Table 1; Supplementary Tables 1–2]. CAPA incidence was 5% (9/190; range: 0%–12.5%) at 4 centers enrolling ≥25 patients, and 10% (11/110) among patients in whom BAL samples were tested. The median days from ICU admission to CAPA diagnosis was 9 (interquartile range: 3 to 17) [Supplementary Figure 1]. Tracheobronchitis was not identified. CAPA was diagnosed by positive BAL GM-EIA (13% of patients tested, 8/60), BAL culture (7%, 6/83), and/or serum GM-EIA (5%, 5/110). Positive BAL cultures recovered *A fumigatus* (n = 4), non-*fumigatus Aspergillus* (n = 1), and *Aspergillus terreus* (n = 1). In 93% (13/14) of cases, ≥ 2 different diagnostic tests were performed; in 38% (5/13) of these cases, ≥ 2 different tests were positive. Including repeated tests, a mean of 2.6 samples (range: 1–5) per CAPA patient were tested by an assay included in MSGERC criteria. Five percent (6/111) of patients with NBL/tracheal aspirate performed had positive cultures for *Aspergillus* spp. Three of these 6 patients subsequently underwent bronchoscopy, and the diagnosis of probable MSGERC CAPA was confirmed by positive BAL culture; 2 patients also had positive serum GM, 1 of whom also had positive BAL GM [Supplementary Table 1]. In the remaining 3 patients, bronchoscopy was not performed. Two of these patients fulfilled the PHW-MRC definition of CAPA (but not MSGCRC or ECMM-ISHAM), and the third patient did not fulfill any of the CAPA definitions. The 2 patients with CAPA by PHW-MRC criteria were treated with an antifungal but died from chronic respiratory failure; the last patient was not treated with an antifungal and survived

Factors associated with CAPA by univariate analysis were requirement for hemodialysis on ICU admission, EORTC/MSGERC host factor, and presence of cavitary lesion on chest radiograph [Table 1]. By multivariable binary logistic regression, factors independently associated with CAPA were cavitary lung lesion (*P* = .002; odds ratio [OR]: 13.1; 95% confidence interval [CI], 2.6–67.3) and EORTC/MSGERC host factor (*P* = .0015; OR: 6.2; 95% CI, 1.7–22.2).

Outcomes. Cumulative incidence of death at 90 days among patients with and without CAPA was 71% (10/14) and 54% (106/197), respectively (*P* = .24). Corresponding incidence rates of death were 2.0 and 1.4/100-patient-days (*P* = .27). Median time from CAPA diagnosis to death was 9 days (range: 5–53). Nine patients died despite antifungal therapy (1 had autopsy-proven IPA) [Supplementary Figure 2A]. A tenth patient died without evidence of IPA on autopsy 13 days after completing a 6-day course of posaconazole. Among 4 survivors, 3 received mold-active antifungal (14, 22, 38 days) and 1 did not receive antifungal treatment.

**Figure 2.**
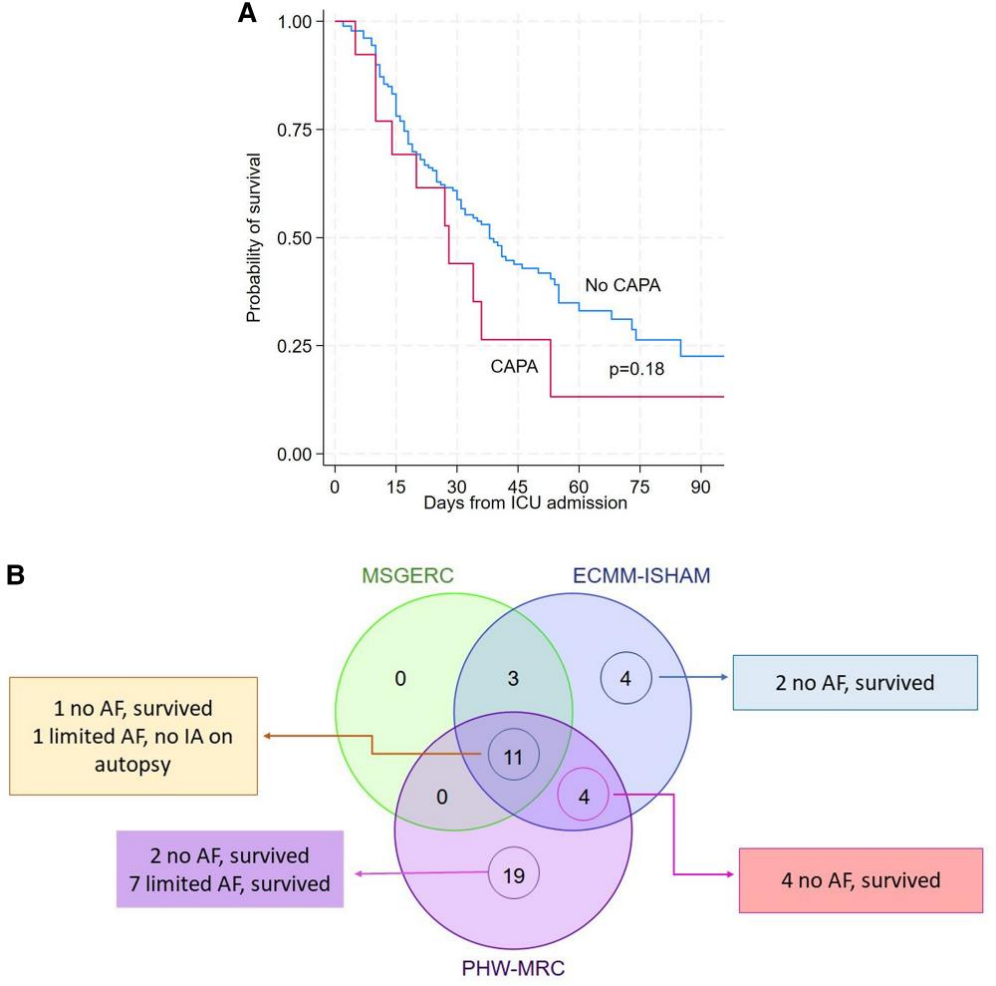
Outcomes of patients with CAPA. (A) Probability of survival stratified by presence or absence of CAPA. Logistic regression analysis did not reveal significant difference in mortality between the 2 groups (log-rank *P* = .18). (B) Number of cases with unlikely IPA among patients with CAPA diagnosed by MSGERC, ECMM-ISHAM, and PHW-MRC definitions. Numbers of patients fulfilling various CAPA definitions are shown within the Venn diagram. Numbers of patients who survived or had negative autopsy findings despite no fungal treatment or limited antifungal treatment (≤10 d) are presented in boxes next to the Venn diagram. These patients were unlikely to have IPA. Fourteen percent (2/14), 36% (8/22), and 44% (15/34) of patients with MSGERC, ECMM-ISHAM, and PHW-MRC CAPA criteria, respectively, either had no or unlikely IPA. Abbreviations: AF, antifungal treatment; CAPA, COVID-19–associated pulmonary aspergillosis; ECMM-ISHAM, European Confederation of Medical Mycology-International Society of Human and Animal Mycology; IPA, invasive aspergillosis; MSGERC, Mycoses Study Group; PHW-MRC, Public Health Wales-Mycoses Research Council.

Older age, EORTC/MSGERC host factor, diabetes mellitus, and hemodialysis were associated with death among critically ill patients with COVID-19 by univariate analysis, whereas CAPA was not [Table 2; Figure 2*A*, log-rank *P* = .18]. Cox multiple regression analysis identified both age and diabetes as independent factors for mortality.

**Table 2.** Risk Factors for Death Among Critically Ill, Mechanically Ventilated Patients With COVID-19

Characteristics	Univariate Analysis			
	Patients who Died (n = 116)^a^	Patients who Lived (n = 95)^a^	*P* Values^b^	Cox Regression Multivariable Analysis^c^ *P* Values Hazard Ratio; 95% Confidence Interval
Men	60% (70/116)	55% (57/94)*	1.0	
Age, median (IQR), y	49 (27)	47 (28)	.0001	*P* = .001 HR: 1.02; 95% CI, 1.10–1.04
Age > 50 y	71% (82/115)*	39% (37/94)*	.0001	
White race	71% (71/100)*	77% (63/82)*	.22	
BMI, median (IQR)	30.6 (8.9)	35.1 (4.6)	.13	
Chronic obstructive pulmonary disease	9% (11)	15% (14)	.29	
Liver cirrhosis	3% (3)	1% (1)	.6	
Diabetes mellitus	45% (52)	21% (20)	<.0001	*P* = .02 HR = 1.16; 95% CI, 1.1–2.5
Diabetic ketoacidosis	9% (6/68)*	2% (2/94)*	0.07	
Hemodialysis on ICU admission	22% (25)	6% (6)	.002	NS
EORTC-MSGERC host factors	32% (37)	11% (11)	.0001	NS
Solid organ transplant	25% (29)	7% (7)	.001	
Neutropenia >10 d	2% (2/106)*	1% (1/87)*	1.0	
Cavitary lung lesion	6% (6)	4% (4)	.76	
MSGERC-defined CAPA	0% (10)	4% (4)	.27	
ECMM-ISHAM-defined CAPA	10% (12/114)	10% (10/95)	.66	
PHW-MRC-defined CAPA	13% (15)	20% (19)	.419	

Abbreviations: 95% CI, 95% confidence interval; BMI, body mass index; CAPA, COVID-19–associated pulmonary aspergillosis; ECMM-ISHAM, European Confederation of Medical Mycology-International Society of Human and Animal Mycology; EORTC-MSGERC, European Organization for Research and Treatment of Cancer-Mycosis Study Group Education and Research Consortium; HR, hazard ratio; ICU, intensive care unit; IQR, interquartile range; NS, not significant; PHW-MRC, Public Health Wales-Mycoses Research Council.

^a^Outcome at 90 d was not known in 1 patient.

^b^ *P* values were calculated using Fisher exact test for categorical variables and Kruskal-Wallis rank test for continuous variables.

^c^Factors identified to be associated with 90-day mortality by Fisher exact or Mann–Whitney *U* tests were further analyzed by Cox regression multivariable analyses with *P* values, HR, and 95% CI as shown in the last column. The Cox proportional hazard model for patients with and without CAPA includes CAPA as a time-dependent variable.

Mortality of MSGERC, ECMM-ISHAM, and PWH-MRC CAPA criteria were 71% (10/14), 50% (11/22), and 44% (15/211), respectively.

^*^Missing data.

Eighty-six percent (12/14) of CAPA patients met clinical definitions for proven (n = 1, positive autopsy) or putative IPA (n = 11, treated with mold-active antifungal and biopsies or autopsies were not attained to prove or disprove IPA) [Figure 1]. Patients with CAPA-proven IPA and CAPA-putative IPA constituted 6% (12/212) of the critically ill COVID-19 cohort. The remaining 14% (2/14) of CAPA patients were unlikely to have IPA, including those described previously who (1) survived 3 months without antifungal treatment and (2) received only 6 days of posaconazole and had no IPA on autopsy.

Estimated IPA likelihoods. Median IPA likelihood in patients with CAPA was 30% (range: 1%–99%), based on combined BAL culture, BAL GM-EIA, and serum GM-EIA results [Table 3]. Estimated likelihoods were 84%, 7%–99%, and 1%–8% in CAPA-proven IPA, CAPA-putative IPA, and CAPA-unlikely IPA, respectively. Including results from *Aspergillus* PCR (AsperGenius) and GM-LFA (IMMY), non–Food and Drug Administration-cleared tests that are not included in MSGERC CAPA definitions, estimated IPA probabilities were 86%, 2%–98%, and ≤1%, respectively [Table 3]. Likelihoods changed with these tests by > ±10% in only 3 patients, each of whom had CAPA-putative IPA (63% to 23%; 22% to 5%; 40% to 11%).

**Table 3.** Test Results, Estimated Likelihoods of Invasive Pulmonary Aspergillosis, Treatment, and Outcomes in Patients With CAPA

Patient	MSG-ERC Diagnostic Tests (BAL Culture, BAL GM-EIA, Serum GM-EIA)			Other Diagnostic Tests (BAL PCR, GM-LFA)			Treatment	Outcome/Notes
	Positive Tests	Negative Tests	Estimated Likelihood of IPA^a^	Positive Tests	Negative Tests	Estimated Likelihood of IPA^a^		
CAPA-proven IPA^b^								
1093-032	BAL Cx Serum GM-EIA	None	84%	BAL PCR	BAL GM-LFA	83%	VORI, 22 d	Died on treatment Proven IPA at autopsy
CAPA-putative IPA^b^								
1125-002	BAL Cx x2 BAL GM-EIA Serum GM-EIA	None	99%	None sent		99%	VORI and MICA, 22 d	Died on treatment
1125-008	BAL GM-EIA	BAL Cx	11%	None sent		11%	POSA, 3 d then stopped due to low clinical suspicion	Died with persistent MRSA BSI and PNA
1093-035	BAL Cx BAL GM-EIA	None	70%	None sent		70%	VORI and CASP, 3 d	Died on treatment
1093-059	BAL Cx BAL GM-EIA	Serum GM-EIA	63%	None	Serum GM-LFA Serum PCR	23%	VORI, 35 d	Died on treatment
1001-025	Serum GM-EIA	BAL Cx	22%	None	Serum GM-LFA Serum PCR	5%	MICA, 2 d	Died on treatment (CAPA and candidemia diagnosed on the same day)
1093-034	BAL Cx	Serum GM-EIA	40%	None	Serum GM-LFA Serum PCR	11%	POSA, 4 d	Died on treatment
1010-005	Serum GM-EIA	None	38%	None sent		38%	MICA, 1 d	Died on treatment
1080-002	Serum GM-EIA	BAL GM-EIA	7%	None	Serum GM-LFA Serum PCR	1%	MICA, 15 d for presumptive invasive candidiasis	Died 34 d after CAPA diagnosis from cardiac arrest
1009-019	BAL Cx x2 Serum GM-EIA	None	98%	None sent		98%	VORI, 38 d	Survived
1093-039	BAL GM-EIA	Serum GM-EIA	17%	BAL PCR x2	BAL GM-LFA Serum PCR Serum GM-LFA	21%	VORI, 22 d	Survived
1125-006	BAL GM-EIA	BAL Cx Serum GM-EIA	17%	None sent		17%	VORI, 14 d	Survived
CAPA-unlikely IPA^b^								
1093-031	BAL GM-EIA	BAL GM-EIA^c^ BAL Cx Serum GM-EIA	1%	None	BAL GM-LFA x2 BAL PCR x2 Serum GM-LFA Serum PCR	<1%	POSA, stopped after 6 d due to low clinical suspicion	Died 13 d after stopping POSA. Autopsy with no evidence of IPA
1093-43	BAL GM-EIA	BAL Cx Serum GM-EIA	8%	Serum PCR	BAL GM-LFA BAL PCR Serum GM-LFA Serum PCR x2	1%	No antifungal treatment	Survived

Abbreviations: BAL, bronchoalveolar lavage; BSI, bloodstream infection; CAPA, COVID-19–associated pulmonary aspergillosis; CASP, caspofungin; Cx, culture; EIA, enzyme immunoassay; GM, galactomannan; IPA, invasive pulmonary aspergillosis; LFA, lateral flow assay; MICA, micafungin; MRSA, methicillin resistant *Staphylococcus aureus*; PNA, pneumonia; POSA, posaconazole; VORI, voriconazole.

^a^Detailed methods and calculations of estimated IPA likelihoods for each case are provided in Supplementary Methods, respectively.

^b^CAPA-proven IPA, CAPA-putative IP, and CAPA-unlikely IPA are clinical classifications. CAPA-proven IPA was diagnosed in patients fulfilling MSG-ERC definitions of CAPA in whom there was tissue evidence of IPA on biopsy or autopsy. CAPA-putative IPA was diagnosed in patients fulfilling MSG-ERC definitions of CAPA who were treated with mold-active antifungal and biopsies or autopsies were not attained to prove or disprove IPA. CAPA-unlikely IPA was diagnosed in patients fulfilling MSG-ERC definitions of CAPA who survived without antifungal treatment or who had no IPA on autopsy after discontinuing short-course antifungal treatment. These classifications are defined in greater detail in Methods and Figure 1.

^c^BAL GM-EIA repeated ≤7 d after initial test.

CAPA by ECMM-ISHAM and PHW-MRC definitions. Numbers of CAPA cases by different definitions are presented in Supplementary Figures 2. CAPA incidence using ECMM-ISHAM and PHW-MRC definitions was 10% (22/212) and 16% (34/212), respectively. Nineteen percent (41/212) and 5% (11/212) of patients with COVID-19 were diagnosed with CAPA by ≥1 or all 3 criteria, respectively. All 14 patients and 79% (11/14) of patients diagnosed by MSGERC were also diagnosed by ECCM-ISHAM or PHW-MRC, respectively [Supplementary Table 1, Supplementary Figure 2A-B-C].

Management and outcomes of patients fulfilling ECMM-ISHAM and PHW-MRC definitions are summarized in Supplementary Figures 2B-C and Supplementary Table 1. Mortality rates among patients diagnosed by MSGERC, ECMM-ISHAM, and PHW-MRC did not significantly differ [Table 2]. Thirty-six percent (8/22) and 44% (15/34) of patients fulfilling ECMM-ISHAM and PHW-MRC criteria, respectively, had CAPA-unlikely IPA (negative autopsy, n = 1; survival without antifungal treatment, n = 9; survival with limited treatment, n = 7 (median: 2 days [range: 1–7]).

Seventy-five percent (6/8) and 57% (13/23) of patients diagnosed by ECMM-ISHAM or PHW-MRC, respectively, but not diagnosed by MSGERC were unlikely to have IPA. Patients with CAPA-unlikely IPA were diagnosed by *Aspergillus* PCR, GM-LFA, serum β-D-glucan, or NBL culture. In 5 patients, PCR was negative on longitudinal samples within 1–7 days after an initial positive result in absence of treatment (1–3 longitudinal samples/patient; BAL, n = 2 patients; serum, n = 3 patients) [Supplementary Table 1].

## DISCUSSION

The incidence of CAPA among critically ill, mechanically ventilated patients with COVID-19 in US ICUs was 7% for the entire cohort and 10% for patients in whom BAL samples were tested by MSGERC criteria that include BAL culture, BAL GM-EIA, and/or serum GM-EIA results. Seven percent (1/14) and 79% (11/14) of CAPA cases met clinical definitions of CAPA-proven IPA and CAPA-putative IPA, respectively. The remaining 14% (2/14) were classified as CAPA-unlikely IPA due to long-term survival without antifungal treatment or a negative autopsy after short-course treatment. Based on diagnostic test sensitivity and specificity derived from published autopsy data, estimated IPA likelihoods were 84% in the patient with proven IPA, 7%–99% in patients with putative IPA, and 1%–8% in those unlikely to have IPA. The wide range of likelihoods with putative IPA is consistent with CAPA being an entity that spans invasive disease and colonization, and with the limited specificity of diagnostic criteria to distinguish along that continuum. Mortality among patients with CAPA was high (71%) despite antifungal treatment. Because antemortem histopathologic proof of IPA (n = 1) and postmortem examinations (n = 2) was uncommon, we cannot definitively determine how often *Aspergillus* infections were invasive or contributed to death. Although data do not resolve ongoing controversies over the incidence or direct impact of CAPA-IPA, this study is important as the first to prospectively describe epidemiology, clinical characteristics, and outcomes of CAPA in multiple centers in North America, and the first to estimate IPA likelihood.

CAPA incidence was 10% (22/212) using ECCM-ISHAM definitions, which are common in European studies and accept PCR and GM-LFA results. Incidence increased to 16% (34/212) with PHW-MRC definitions, which also include serum BDG and NBL culture. All cases identified by MSGERC were identified by ECCM-ISHAM and 79% (11/14) were identified by PHW-MRC. In contrast, 18% (4/22) of ECCM-ISHAM and 56% (19/34) of PHW-MRC cases were diagnosed solely by that definition. Seventy-five percent (6/8) and 57% (13/23) of patients diagnosed by ECMM-ISHAM or PHW-MRC, respectively, but not by MSGERC, survived with no or limited antifungal treatment, indicating that they were unlikely to have IPA. In 5 of these patients, an initial positive *Aspergillus* PCR was followed within 1–7 days by negative results from longitudinal samples. Findings support concerns about *Aspergillus* PCR false positivity for CAPA [16], even using a more conservative cutoff (C_T_ = 32) than proposed by ECCM-ISHAM (C_T_ = 36) or PHW-MRC (detectable signal). A single BAL GM-EIA was likely false positive in 2 patients diagnosed by all 3 criteria who received limited antifungal treatment and either survived or had no evidence of IPA at autopsy. In a study of mechanically ventilated patients with COVID-19, most positive BAL GM-EIA or culture results were not repeatable on testing a second sample [4]. Data on serum GM-LFA, BDG, or NBL cultures are too limited here to draw definitive conclusions on performance. Until more data are available, we recommend that clinicians use and interpret *Aspergillus* PCR, serum GM-LFA and BDG, and NBL cultures with caution.

Repeated testing by a given assay or combination testing with different assays has been proposed to improve CAPA diagnoses by increasing specificity and predictive value for IPA if multiple results are positive [5, 12]. Concordant positive or negative results are powerful markers for disease presence or absence because the final likelihood ratio is the product of individual test likelihood ratios. However, our data reenforce an often-overlooked feature of repeated or combination testing: discordant results counteract one another. CAPA is overdiagnosed by accepting a positive result without considering negative results from that test or others.

Many of our findings are consistent with those of previous studies. CAPA-proven or CAPA-putative IPA were diagnosed by clinical criteria in 6% (12/212) of patients with severe COVID-19. Given that some putative CAPA-IPA diagnoses were likely false positive, results are in general keeping with 2% incidence of invasive mould infections in a systematic review of published autopsies of mechanically ventilated patients with COVID-19 in the first year of the pandemic [7]. Patient demographics, clinical features, time to diagnoses, and high mortality rates here are similar to data from other CAPA studies. CAPA was not a risk factor for death among patients with COVID-19, consistent with findings of some, but not all previous reports [1]. As in our experience, studies often show a wide range of CAPA incidence at different hospitals. Explanations for variation between centers are unclear but could include differences in case definitions, local fungal ecology, at-risk patient populations, thresholds for testing, frequency of testing, and socioeconomic factors.

Roles of antifungal treatment or prophylaxis in patients with CAPA or COVID-19, respectively, are not established. As shown here, there is no conclusive evidence that antifungal treatment of CAPA improves outcomes. In several observational studies, antifungal prophylaxis of severe COVID-19 was associated with reductions in CAPA diagnoses, but not with improved survival [17–19]. Lacking definitive clinical trial data and given poor overall patient outcomes, clinicians may decide that antifungal treatment of CAPA is justifiable at some, locally agreed-on threshold probability of IPA. The key to managing CAPA is maintaining a level of suspicion to initiate diagnostic workup in at-risk populations with severe COVID-19. Absent tissue diagnosis of IPA, antifungal treatment can be initiated in these patients on positive BAL culture, BAL GM-EIA, or serum GM-EIA. Thereafter, repeating these tests on concurrent or longitudinal samples can guide continuation or discontinuation decisions. Based our findings, there was limited utility of *Aspergillus* PCR or other non–Food and Drug Administration-cleared assays beyond that provided by BAL culture and BAL or serum GM-EIA. A strength of this study was its prospective enrollment of patients from hospitals in various US geographic regions, using protocols designed by and conducted under auspices of MSGERC. Systematic screening for CAPA was not performed at any center and workup and management decisions were left solely to local clinicians’ discretion. Because clinicians often chose not to treat patients who fulfilled at least some CAPA criteria, we were able to study diagnostic test results in many patients who survived in absence of significant antifungal exposure. In other ways, heterogeneity in diagnostic and management practices and small number of autopsies were shortcomings that limited our ability to more precisely define incidence or impact of treatment.

In conclusion, there is pressing need for a global study of CAPA and other severe viral infection–associated aspergillosis using standardized and uniform methodology. Single positive diagnostic test results are not sufficient for identifying CAPA-associated IPA. Both positive and negative test results must be considered along with clinical features in estimating disease likelihood in individual cases. With the close of the public health emergency phase of the COVID-19 pandemic, insights from this study may prove most valuable in the future in considering how to best manage and investigate aspergillosis in critically ill respiratory failure populations that lack traditional immunosuppression-associated risk factors.

## Supplementary Material

ofaf331_Supplementary_Data

## Supplementary Data

Supplementary materials are available at *Open Forum Infectious Diseases* online. Consisting of data provided by the authors to benefit the reader, the posted materials are not copyedited and are the sole responsibility of the authors, so questions or comments should be addressed to the corresponding author.

## Notes

***Acknowledgments.*** The authors acknowledge members of the research team (Anthony Newbrough, Wendy Liang, Carolyn Grimes, Virginia Umana, Mehriban Mammadova, Jessica Sanchez, Maria Hernandez) and the UAB Fungal Reference Laboratory (Derek Moates, Jeremy Meeder) who contributed to the successful completion of the study.

***Financial support.*** This work was supported by The Centers for Disease Control (grant No 75D30120C09617) and Astellas Pharma US (CRES-20H01)
