# Supplementary material for: COVID-19-associated Pulmonary Aspergillosis in Mechanically Ventilated Patients at 7 US Hospitals: Epidemiology and Estimated Likelihood of Invasive Pulmonary Aspergillosis—Results of the Prospective MSG-017 Study
Source: Open Forum Infect Dis. 2025 Jul 17;12(7):ofaf331. doi: 10.1093/ofid/ofaf331 (PMC12268869; doi:10.1093/ofid/ofaf331)
Supplement: ofaf331_Supplementary_Data [file ofaf331_supplementary_data.zip › CAPA Supplementary Figures OFID.docx]

**Supplementary Figures**

**Supplementary Figure 1. Time from ICU admission to MSGERC CAPA diagnosis.**

To estimate time to event, cumulative incidence function was performed to account for death as competing event.

**Supplementary Figure 2. Management and outcomes of CAPA diagnosed by MSGERC (2a), ECMM-ISHAM (2b) and PHW-MRC (2c) definitions.**


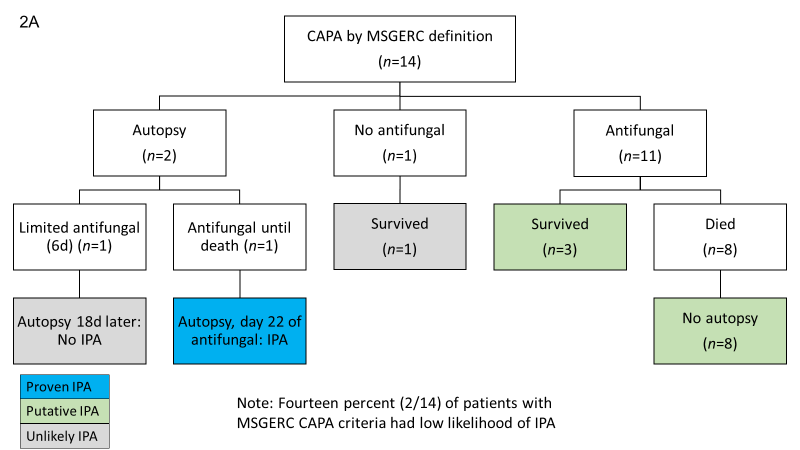


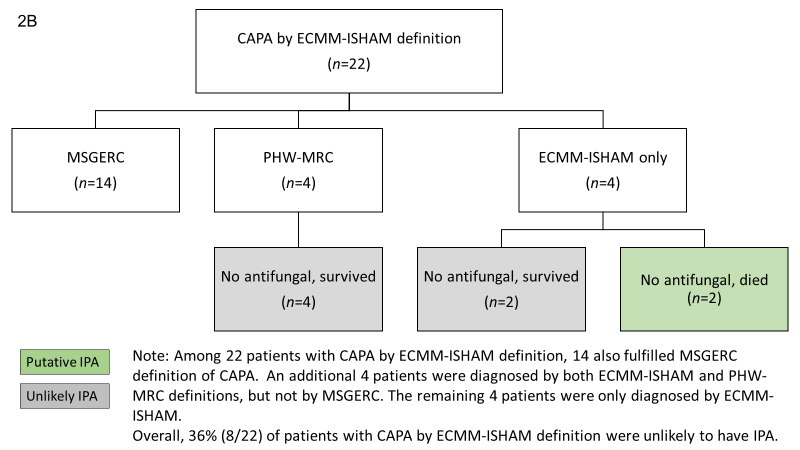


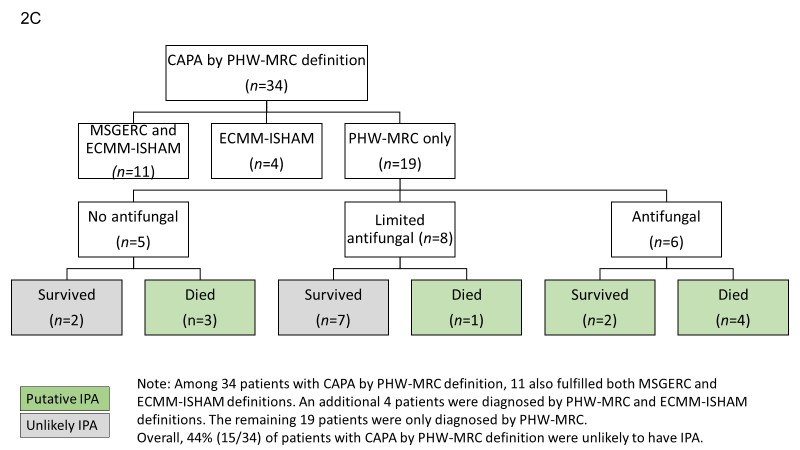


The blue box (Figure 2a) denotes proven IPA. Green boxes (Figure 2a, 2b, 2c) denote probable IPA. Grey boxes (Figures 2a, 2b, 2c) denote low likelihood of IPA because of no evidence for invasive fungal infection on autopsy, or survival despite no or limited (<10 days) antifungal treatment.
