# Supplementary material for: COVID-19-associated Pulmonary Aspergillosis in Mechanically Ventilated Patients at 7 US Hospitals: Epidemiology and Estimated Likelihood of Invasive Pulmonary Aspergillosis—Results of the Prospective MSG-017 Study
Source: Open Forum Infect Dis. 2025 Jul 17;12(7):ofaf331. doi: 10.1093/ofid/ofaf331 (PMC12268869; doi:10.1093/ofid/ofaf331)
Supplement: ofaf331_Supplementary_Data [file ofaf331_supplementary_data.zip › CAPA supplementary Methods FINAL CID.docx]

**SUPPLEMENTAL METHODS.**

**Supplemental Methods 1.** Criteria in PHW-MRC and ECMM-MRC definitions of COVID-19 associated pulmonary aspergillosis (CAPA) are summarized in the following table (adapted from ^1^)

| **Definition** | | **Clinical and Host Factors** | **Radiologic Findings** | **Mycologic Criteria** |
| --- | --- | --- | --- | --- |
|  | **ECCM-ISHAM^2^**  **Proven CAPA** | COVID-19 positive patient needing intensive care and temporal relationship with signs and symptoms of worsening respiratory status not explained by other diagnoses |  | 1) Histopathologic or direct microscopic detection of fungal hyphae showing invasive growth with associated tissue damage  AND/OR  2) *Aspergillus* spp. recovered by culture or microscopy/histology or PCR obtained by a sterile aspiration or biopsy from a pulmonary site showing an infectious disease |
|  | **ECCM-ISHAM^2^**  **Probable CAPA** | COVID-19 positive patient needing intensive care and temporal relationship with signs and symptoms of worsening respiratory status not explained by other diagnoses | Pulmonary infiltrate, preferable documented by chest CT  OR  Cavitating infiltrate (not attributed to another cause) | AND ≥1 of the following positive:  1) Microscopic detection of fungal elements in BAL indicating a mould  2) Positive BAL culture or PCR  3) Serum GM ≥ 0.5 ODI or Serum LFA index ≥ 0.5  4) BAL GM ≥ 1.0 ODI or BAL LFA index ≥ 1.0  5) Multiple (≥ 2) positive *Aspergillus* PCR in plasma, serum or whole blood  6) Single positive *Aspergillus* PCR in BAL fluid (36 cycles)  7) Single positive *Aspergillus* PCR in plasma, serum or whole blood and a single positive BAL fluid (any C_T_ permitted) |
|  | **PHW-MRC^3^**  **Proven CAPA** | PCR-confirmed COVID-19 infection and 1 of:  1) Refractory fever despite ≥ 3 days antibiotics  2) Recrudescent fever of at least 48 hours despite antibiotics  3) Dyspnea  4) Hemoptysis  5) Pleural rub or chest pain  6) Worsening respiratory function despite antibiotics and ventilatory support | New infiltrates on chest x-ray or chest CT when compared to admission, including progression of signs attributed to viral infection. Radiological signs typical of invasive pulmonary aspergillosis (nodules, halos, cavities, wedge-shaped and segmental or lobar consolidation) or evidence of sinusitis should be associated with heightened suspicion of fungal disease | 1) Histology/microscopy demonstrating dichotomous septate hyphae in tissue  2) Positive culture from tissue |
|  | **PHW-MRC^3^**  **Putative CAPA, Non-Specific Radiology** | PCR confirmed COVID-19 infection and 1 of:  1) Refractory fever despite ≥ 3 days antibiotics  2) Recrudescent fever of ≥48 hours despite antibiotics  3) Dyspnea  4) Hemoptysis  5) Pleural rub or chest pain  6) Worsening respiratory function despite antibiotics and ventilatory support | Non-specific radiology or radiology typical of IA attributed to a differing underlying infection (e.g., lung cancer or alternative infection) | ≥ 2 positives across different test types, or multiple positives within a single test type from the following:  1) Positive culture from NBL/BAL  2) Positive GM in NBL/BAL (≥ 1.0 ODI)  3) Positive GM in serum (≥ 0.5 ODI)  4) Positive *Aspergillus* PCR in BAL or blood  5) Positive BDG in serum/plasma |
|  | **PHW-MRC^3^**  **Putative CAPA, Radiology Typical of IPA** | PCR confirmed COVID-19 infection and 1 of:  1) Refractory fever despite ≥ 3 days antibiotics  2) Recrudescent fever of ≥48 hours despite antibiotics  3) Dyspnea  4) Hemoptysis  5) Pleural rub or chest pain  6) Worsening respiratory function despite antibiotics and ventilatory support | Radiology typical of IA: New infiltrates on chest x-ray or chest CT when compared to admission, including progression of signs attributed to viral infection. Radiological signs typical of invasive pulmonary aspergillosis (nodules, halos, cavities, wedge-shaped and segmental or lobar consolidation) or evidence of sinusitis should be associated with heightened suspicion of fungal disease | ≥ 1 of the following positive:  1) Positive culture from NBL/BAL  2) Positive GM in NBL/BAL (≥  1.0 ODI)  3) Positive GM in serum (≥  0.5 ODI)  4) Positive *Aspergillus* PCR in BAL or blood  5) Positive BDG in serum/plasma |

Abbreviations: BAL: bronchoalveolar lavage; BDG: 1-3-β-D-glucan; CAPA: COVID-19 associated pulmonary aspergillosis; CT: computed tomography; ECMM-ISHAM: European Confederation of Medical Mycology-International Society for Human and Animal Mycoses; GM: galactomannan; HIV: human immunodeficiency virus HSCT: hematopoetic stem cell transplant; IA: invasive aspergillosis; IAPA: influenza-associated pulmonary aspergillosis; LFA: lateral flow assay; mTOR: mammalian target of rapamycin; NBL: non-bronchoscopic lavage; ODI: optical density index; PCR: polymerase chain reaction; SOT: solid organ transplant TNF: tumor necrosis factor.

**Supplemental Methods 2.** Estimation of IPA likelihood among patients with CAPA by MSGERC definition.

Estimated IPA was calculated as follows:

**Likelihood of IPA with positive test = (Sensitivity x pre-test likelihood of IPA) / (Sensitivity x pre-test likelihood of IPA) + ((1 - specificity) x (1 - pre-test likelihood of IPA)).**

**Likelihood of IPA with negative test = 1- (Specificity x (1 – pre-test likelihood of IPA)) / (Specificity x (1 – pre-test likelihood of IPA)) + ((1 – sensitivity) x pre-test likelihood of IPA)**

Sensitivity and specificity (i.e., performance) of tests for diagnosing IPA was taken from published data. For BAL culture, BAL GM-EIA and serum GM-EIA, performance was from data on autopsy-proven IPA in patients with pre-mortem diagnosis of CAPA.^4^ For *Aspergillus* PCR and GM-LFA, there are limited data on performance in diagnosing IPA in patients with COVID-19. Therefore, for *Aspergillus* PCR, we used performance of the AsperGenius assay in various hosts with EORTC/MSGERC risk factors for aspergillosis, as published for testing BAL^5,6^ and plasma.^7^ For GM-LFA, clinical performance data are also limited, but agreement with GM-EIA results in respiratory samples was reported as excellent. Therefore, we used performance of GM-EIA in estimating IPA likelihood based on GM-LFA results. The table below summarizes performance assigned to each test.

| **Test** | **Sensitivity** | **Specificity** | **Comments and Reference** |
| --- | --- | --- | --- |
| BAL GM-EIA | 92% | 64% | Based on autopsy data for IPA among patients diagnosed with CAPA pre-mortem.^4^ |
| Serum GM-EIA | 33% | 94% |  |
| BAL culture | 58% | 93% |  |
|  | | | |
| BAL PCR | 84% | 89% | References from Chong^5^ and Schauwvlieghe^6^ |
| Serum PCR | 80% | 78% | Reference from White et al^7^ |
| BAL GM-LFA | 92% | 64% | Assumptions based on GM-EIA |
| Serum GM-LFA | 33% | 94% | Assumptions based on GM-EIA |

In each case, we began our calculations with one test result by taking pre-test likelihood of 10%, based on median prevalence of CAPA from studies cited in the literature.^1^ The calculated likelihood was then used as the pre-test likelihood for the next test result. This process was repeated for all test results in the given case to arrive at the final estimated likelihood. Calculations for each patient diagnosed with CAPA are show Data file (Excel file).

**References.**

1. Kariyawasam RM, Dingle TC, Kula BE, Vandermeer B, Sligl WI, Schwartz IS. Defining COVID-19-associated pulmonary aspergillosis: systematic review and meta-analysis. Clin Microbiol Infect 2022;28:920-7.

2. Koehler P, Cornely OA, Bottiger BW, et al. COVID-19 associated pulmonary aspergillosis. Mycoses 2020;63:528-34.

3. White PL, Dhillon R, Cordey A, et al. A national strategy to diagnose COVID-19 associated invasive fungal disease in the ICU. Clin Infect Dis 2020.

4. Vanderbeke L, Jacobs C, Feys S, et al. A Pathology-based Case Series of Influenza- and COVID-19-associated Pulmonary Aspergillosis: The Proof Is in the Tissue. Am J Respir Crit Care Med 2023;208:301-11.

5. Chong GM, van der Beek MT, von dem Borne PA, et al. PCR-based detection of Aspergillus fumigatus Cyp51A mutations on bronchoalveolar lavage: a multicentre validation of the AsperGenius assay(R) in 201 patients with haematological disease suspected for invasive aspergillosis. J Antimicrob Chemother 2016;71:3528-35.

6. Schauwvlieghe A, Vonk AG, Buddingh EP, et al. Detection of azole-susceptible and azole-resistant Aspergillus coinfection by cyp51A PCR amplicon melting curve analysis. J Antimicrob Chemother 2017;72:3047-50.

7. White PL, Posso RB, Barnes RA. Analytical and Clinical Evaluation of the PathoNostics AsperGenius Assay for Detection of Invasive Aspergillosis and Resistance to Azole Antifungal Drugs Directly from Plasma Samples. J Clin Microbiol 2017;55:2356-66.
